# Cardiovascular complications and predictors of mortality in hospitalized patients with COVID-19: a cross-sectional study from the Indian subcontinent

**DOI:** 10.1186/s41182-022-00481-w

**Published:** 2022-12-19

**Authors:** Kanhai Lalani, M. Sudhakar Rao

**Affiliations:** Department of Cardiology, Kasturba Medical College, Manipal, Manipal Academy of Higher Education, Manipal, Karnataka 576104 India

**Keywords:** COVID-19, Coronavirus, Severe acute respiratory syndrome coronavirus 2 (SARS‑CoV‑2), Cardiovascular complications, Myocarditis, Acute coronary syndrome, Neutrophil–lymphocyte ratio (NLR), Platelet–lymphocyte ratio (PLR)

## To the editor

We gratefully acknowledge Dr. Josef Finsterer's insightful comments on our study, “Cardiovascular complications and predictors of mortality in hospitalized patients with COVID-19: a cross-sectional study from the Indian subcontinent” [1]. We hope to address some of the concerns raised by J. Finster in this response.

Their first concern was the study's retrospective design. We acknowledge their concern and have addressed it in the limitations section of this study [1]. We have collected the data of COVID-19 patients admitted to our hospital during the first wave of COVID-19 in our country, before which no one had predicted such a pandemic [1]. We carefully observed certain hemocytometric parameters, electrocardiographic (ECG) changes, and cardiac complications among COVID-19 patients, and decided to publish a study on them after receiving ethics committee approval [1]. Hence, we conducted a retrospective study. However, all the blood investigations and ECGs were done by the same machine for all the patients included at our hospital. The echocardiogram, on the other hand, was performed by the same machine but by different operators, which was a limitation for us. Ideally, we should conduct a prospective study to overcome all the shortcomings.

Their second concern was that the diagnosis of myocardial infarction was based solely on clinical and ECG criteria, rather than on coronary angiography (CAG) findings. 9 (64.3%) of 14 patients with anterior wall myocardial infarction (AWMI) underwent CAG and percutaneous coronary intervention (PCI) out of a total of 67 patients who presented with the acute coronary syndrome (ACS). 9 (75.0%) of 12 patients with inferior wall myocardial infarction (IWMI) or infero-posterior wall myocardial infarction (IPWMI) underwent CAG and PCI. 4 (9.8%) of 41 patients with non-ST-segment elevation myocardial infarction (NSTEMI) underwent CAG and PCI due to angina. The remaining patients were either thrombolysed or managed medically (Table 1). The treating physician or cardiologist determined which patients required invasive management.

**Table 1:** The number of COVID-19 patients who had ACS and underwent CAG and PCI

Complications	Total (*n* = 730)	CAG and PCI (*n* = 22)
ACS (*n*, %)	67 (9.2%)	
AWMI	14 (20.9%)	9 (64.3%)
IWMI	12 (17.9%)	9 (75.0%)
NSTEMI	41 (61.2%)	4 (9.8%)

Their third concern was that Takotsubo syndrome (TTS) was not considered as a complication of COVID-19. However, a coronary angiogram (CAG) is required to rule out significant coronary artery disease before diagnosing TTS [2]. During the beginning of the pandemic in our country, no standard government protocol was published for the management of ST-segment elevation myocardial infarction (STEMI)/NSTEMI. According to local hospital policy, only a few ACS patients with COVID-19 underwent CAG and PCI with strict precautions, while the rest were kept on medical management to prevent virus spread. The treating physician or cardiologist determined which patients required invasive management. Patients who had ACS but did not have CAG and were treated medically were more likely to have stress cardiomyopathy or Takotsubo syndrome (TTS). All patients with elevated troponin could not undergo CAG due to multiple restrictions imposed by the government and local hospital policies to limit the spread and avoid unnecessary exposure to COVID-19. As a result, we did not mention TTS as a COVID-19 complication.

Their fourth concern was that endocarditis was not mentioned as a cardiac complication of COVID-19, which had recently been reported in a few case reports [3–5]. However, patients with preexisting cardiac conditions, including prosthetic valves and implanted devices, were already excluded from our study [1]. We enrolled patients in our study at the beginning of the pandemic in our country, so they were exposed to COVID-19 for the first time with no prior exposure [1]. The case report published by Miri et al. [3] has clearly mentioned that the patient had COVID-19 1 month prior and recent RT-PCR was negative during the second admission with infective endocarditis. The second case reported by Romeo et al. [4] had a history of prior cardiac surgery, and he was treated with corticosteroids, long broad-spectrum antibiotics, and immunosuppressors during his prolonged ICU stay with COVID-19 illness; he was later readmitted with a fungal infection. Vivo et al. [5] reported a third case with preexisting cardiac disease and an implanted device in situ. He had COVID-19 pneumonia as well as *Staphylococcus epidermidis*-positive infective endocarditis at the same time. However, no conclusive evidence linking COVID-19 and endocarditis has been reported. Patients with a preexisting cardiac condition or a history of COVID-19 illness were excluded from our study. As a result, no patients with prosthetic valves or implanted devices were included in the study.

Their fifth concern was whether myocarditis should be diagnosed using cardiac magnetic resonance imaging (MRI) or endo-myocardial biopsy. We all agree that a cardiac MRI or biopsy should be used to confirm a diagnosis of myocarditis. However, due to local hospital policy and limited resources in developing countries, cardiac MRI or biopsy was not possible in our study populations. During the pandemic, only life-saving procedures were permitted in order to limit the spread of COVID-19. Due to limited resources, a large (1791) number of patients were unable to obtain an echocardiogram and were thus excluded from the study [1]. As a result, myocarditis was diagnosed solely on the basis of echocardiographic findings, which may have underestimated many subclinical cases, which was a major limitation in our study and was mentioned in the limitations.

Overall, this interesting article has several limitations which challenge our results and interpretation. We hope that our clarifications to your responses have been adequately addressed, and that the disease under study requires a larger prospective study in the future to overcome these limitations. We want to thank our colleagues once more for their interest in our research.

## Data Availability

The datasets used and/or analyzed during the current study are available from the corresponding author upon reasonable request.
